# NADPH‐Related Enzymes and Cancer: Facts and Insights Into the Application of Immunohistochemistry

**DOI:** 10.1155/bri/1853546

**Published:** 2026-02-16

**Authors:** Camila M. Scudeler, Keila Samantha da Silva, Rafaela V. N. Silva, Adilha M. R. Micheletti, Karen B. Ribeiro, Juliana Reis Machado, Régia C. P. Lira

**Affiliations:** ^1^ Department of Pathology, Genetics and Evolution, Federal University of Triângulo Mineiro, Uberaba, Minas Gerais, Brazil, uftm.edu.br; ^2^ Pelvic Floor Functional Assessment Lab–LAFAP of Ribeirão Preto Medical School, University of São Paulo, Ribeirão Preto, São Paulo, Brazil, usp.br; ^3^ Department of Gynecology and Obstetrics of Ribeirão Preto Medical School, University of São Paulo, Ribeirão Preto, São Paulo, Brazil, usp.br; ^4^ Kidney Research Center, Federal University of Triângulo Mineiro, Uberaba, Minas Gerais, Brazil, uftm.edu.br; ^5^ Department of Surgical Clinic, Federal University of Triângulo Mineiro, Uberaba, Minas Gerais, Brazil, uftm.edu.br; ^6^ Department of Clinical Oncology, Federal University of Triângulo Mineiro, Uberaba, Minas Gerais, Brazil, uftm.edu.br

**Keywords:** cancer, enzymes, immunohistochemistry, NADPH

## Abstract

During tumorigenesis and metastasis, cancer cells initiate antioxidant defense mechanisms to prevent irreversible damage, thereby sustaining tumor growth. The functionality of reactive oxygen species (ROS)–scavenging proteins is dependent on nicotinamide adenine dinucleotide phosphate (NADPH), which is regulated by specific metabolic enzymes, which are described as potential biomarkers of cancer aggressiveness. Immunohistochemistry (IHC) is one of the most accessible and widely utilized techniques to augment the pathological diagnosis of cancer. Hence, this review addresses the protein expression of NADPH‐related enzymes, as assessed by IHC, and their associations with human cancer progression factors (overall survival, tumor staging, metastasis, and recurrence). Studies indicate that glucose‐6‐phosphate dehydrogenase (G6PD), along with malic enzymes and methylenetetrahydrofolate dehydrogenase 2 (MTHFD2), represents the most pertinent enzymes examined through IHC concerning cancer aggressiveness. The immunolabeling method produced consistent results for this group of enzymes, which might lead to successful application in predicting tumor prognosis. Other NADPH‐related enzymes, such as glutamate dehydrogenase (GDH), aldehyde dehydrogenase 1 (ALDH1), and dihydrofolate reductase (DHFR), deserve more extensive investigation to elucidate their potential as cancer biomarkers via IHC.

**Practitioner Points**

• Protein expressions of NADPH‐related enzymes such as G6PD, ME, and methylenetetrahydrofolate dehydrogenase (MTHFD) families suggest cancer aggressiveness due to their associations with tumor size, stage, metastasis, relapse, and/or patient survival.
• In contrast, the enzymes GDH, ALDH1L1, ALDH1L2, and DHFR suggested malignant behavior or a therapeutic response only in a few reports, which suggest the need for more investigations on cancer.
• Immunohistochemistry (IHC) showed consistent results, which directly led to successful protocols for investigating cancer aggressiveness.

## 1. Introduction

According to the World Health Organization (WHO), cancer is a leading cause of premature death worldwide, with more than 10 million casualties annually. The incidence and mortality rates persistently rise because of factors such as population growth, aging, and exposure to risk factors, including obesity, smoking, alcohol consumption, and stress [1, 2]. Studies have endeavored to discover mechanisms inherent to cancer cells’ capability to survive, proliferate, and metastasize under adverse conditions. Most of these findings propose molecular classification and therapeutic targets that hold reliable application in clinical practice [3]. However, the integration of tumor biomarkers into diagnostic routine is still a challenge due to financial and high‐quality professional needs. For this reason, the histopathological features and Immunohistochemistry (IHC) remain the predominant tools used in low‐income countries [4, 5]. Established biomarkers for IHC are crucial for assessing cancer diagnostics, treatment response, and prognosis, offering efficient and cost‐effective morphological analysis [6].

During carcinogenesis and tumor progression, the altered metabolism increases reactive oxygen species (ROS), inducing cytotoxicity, DNA damage, and apoptosis. To avoid lethal damage, cancer cells activate antioxidant defenses, which are dependent on nicotinamide adenine dinucleotide phosphate (NADPH) [7]. The preference of cancer cells for glycolysis under aerobic conditions (Warburg effect) contributes to the maintenance of NADPH, favoring tumor progression [8, 9]. Increasing evidence suggests that expressions of NADPH‐related enzymes are prognostic biomarkers and anticancer therapy targets [10]. NADPH synthesis is driven by enzymes such as malic enzymes (MEs: ME1, ME2, and ME3), glucose‐6‐phosphate dehydrogenase (G6PD), NAD kinases (NADK), phosphogluconate dehydrogenase (PGD), Methylenetetrahydrofolate dehydrogenases (MTHFDs;MTHFD1, MTHFD1L, MTHFD2, and MTHFD2L), aldehyde dehydrogenases (ALDH1L1 and ALDH1L2), isocitrate dehydrogenases (IDH1 and IDH2), dihydrofolate reductase (DHFR), nicotinamide nucleotide transhydrogenase (NNT), and glutamate dehydrogenases (GDHs: GDH1 and GDH2) [7].

Among the NADPH‐related enzymes addressed in this review, G6PD is investigated with notable frequency by IHC in cancer specimens, being the high expression associated with malignancy and poor prognosis (Table 1). Outstanding IHC findings for MTHFD2 and MTHFD1L were also predictive of cancer progression, whereas high expressions of ME Types 1 and 2 suggest breast cancer aggressiveness [44]. Low GDH immunostaining correlated with aggressiveness in primary unknown metastatic cancer [45], pancreatic cancer [47], renal cell carcinoma (RCC) [49], and colorectal cancer [46]. Similarly, diminished expressions of ALDH1L1 and ALDH1L2 predict unfavorable outcomes in hepatocellular, oral, and colorectal cancers [50–52]. DHFR higher expression was associated with osteosarcoma [54] and chondrosarcoma [56] progression without significant results in lung [57, 58], pancreatic [55], and ovarian [53] cancers. Figures 1 and 2 illustrate immunolabeling patterns for the major enzymes, respectively, associated and not associated with malignancy or tumor prognosis. All images were obtained from the Protein Atlas database (https://www.proteinatlas.org) for illustrative purposes; clinical annotations and survival data for the corresponding cases are not available.

**Table 1:** NADPH‐related protein expression patterns in neoplastic tissues and their associations with malignancy and prognosis.

Enzyme	Type of neoplasia	Expression pattern in tumors	Association with malignancy or prognostic factors	Reference
G6PD	Hepatocellular carcinoma	High	Yes^a,b,c^	(Hu et al.) [11]
				(Chen et al.) [12]
				(Song et al.) [13]
	Breast cancer	High	Yes^b^	(Pu et al.) [14]
			Yes	(Dong et al.) [15]
	Clear cell renal cell carcinoma	High	Yes	(Zhang et al.) [16]
			Yes^a,b,c^	(Zhang et al.) [17]
	Gastric cancer	High	Yes	(Wang et al.) [18]
	Esophageal carcinoma	High	Yes	(Wang et al.) [19]
	Cervical cancer (HPV+)	High	Yes^a,c^	(Hu et al.) [20]
	Brain metastases of breast cancer	High	No	(Cha et al.) [21]
	Lung cancer	High	Yes	(Nagashio et al.) [22]
	Oral carcinoma	High	Yes	(Wang et al.) [23]
	Pediatric rhabdomyosarcoma	High	Yes^b^	(Felkai et al.) [24]
	Adrenal neoplasm	High	No	(Kim et al.) [25]
	Pediatric osteosarcoma	Positive	No^b^	(Mohás et al.) [26]

MTHFD1	Hepatocellular carcinoma	High	Yes	(Yu et al.) [27]
	Lung cancer	Positive	No	(Yao et al.) [9]
	Gallbladder carcinoma	Positive	No^b,c^	(Yang et al.) [28]

MTHFD1L	Melanoma	Positive	No	(Byström et al.) [29]
	Esophageal carcinoma	High	Yes^a,b,c^	(Yang et al.) [30]
	Colorectal cancer	High	Yes	(He et al.) [31]
	Hepatocellular carcinoma	High	Yes^a,b,c^	(Chen et al. 2021) [32]

MTHFD2	Breast cancer	High	Yes	(Liu et al.) [33]
	Renal cell carcinoma	High	Yes	(Lin et al.) [34]
				(Silva et al.) [35]
	Esophageal carcinoma	High	Yes	(He et al.) [36]
	Lung cancer	High	Yes	(Yao et al.) [9]
	Ovarian cancer	High	Yes	(Li et al.) [37]
	Bladder cancer	High	Yes	(Deng et al.) [38]

ME1	Oral carcinoma	High	Yes	(Nakashima et al.) [39]
	Gastric cancer	High	Yes	(Shi et al.) [40]
	Breast cancer	High	Yes	(Liu et al.) [41]

ME2	Melanoma	High	Yes	(Chang et al.) [42]
	Salivary glands carcinoma	High	Yes^a,b,c^	(Wu et al.) [43]
	Breast cancer	High	Yes	(You et al.) [44]

GDH (GLUD1)	Primary unknown metastatic carcinoma	Low	Yes^b^	(Kim et al.) [45]
	Colorectal cancer	High	Yes	(Liu et al.) [46]
	Pancreatic cancer	Low	Yes	(Yu et al.) [47]
	Oral carcinoma	Low	No^b,c^	(Cetindis et al.) [48]
	Renal cell carcinoma	Low	Yes^a,b,c^	(Wang et al.) [49]

ALDH1L1	Hepatocellular carcinoma	Low	Yes	(Chen et al.) [50]
	Oral carcinoma	Low	Yes	(Qu et al.) [51]

ALDH1L2	Colorectal cancer	Low	Yes	(Yu et al.) [52]

DHFR	Ovarian cancer	Low	No^b^	(Chen and Li) [53]
	Osteosarcoma	High	Yes	(Scionti et al.) [54]
	Pancreatic cancer	Positive	No	(Chang et al.) [55]
	Chondrosarcoma	High	Yes	(He et al.) [56]
	Lung cancer	High	No	(Chen et al.) [57]
				(Shimokawa et al.) [58]

^a^Significantly higher or lower expression in malignant lesions than in non‐neoplastic or benign tissues.

^b^Low number of samples.

^c^Prognostic factors were not evaluated or showed no significant differences according to the protein expression profile.

Figure 1Protein expression patterns of NADPH‐related enzymes, whose increased expression was associated with malignancy or prognosis in different types of cancer: (a) G6PD, (b) MTHFD2, (c) ME1, and (d) ME2. The immunohistochemistry images were obtained from the Protein Atlas database (https://www.proteinatlas.org) and are shown exclusively for illustrative purposes; clinical annotations and survival data for the corresponding cases are not available. The antibodies (Ab) used were validated and reported by the database, and their reference numbers are indicated below each image. The biomarker is indicated on the left side of the image. The expression was assessed based on the intensity of staining (weak, moderate, and strong) and the number of positive cells (negative, < 25%, 25%–75%, and > 75%), and the expression was classified as high, medium, or low.

**Figure:**
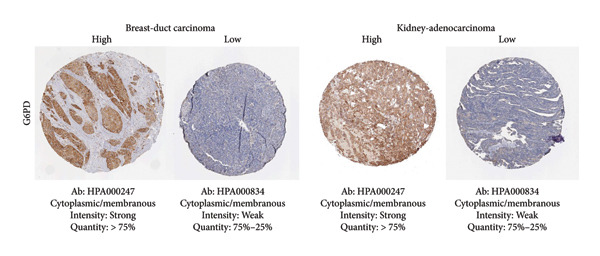
(a)

**Figure:**
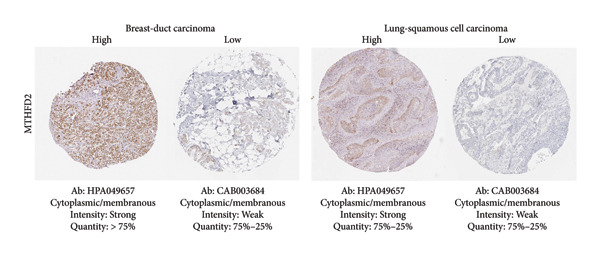
(b)

**Figure:**
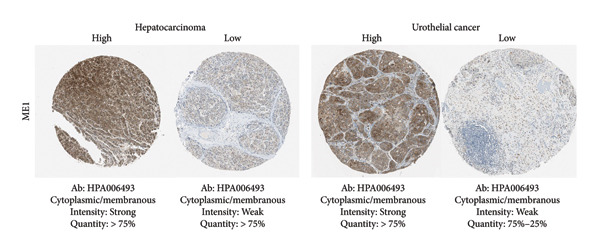
(c)

**Figure:**
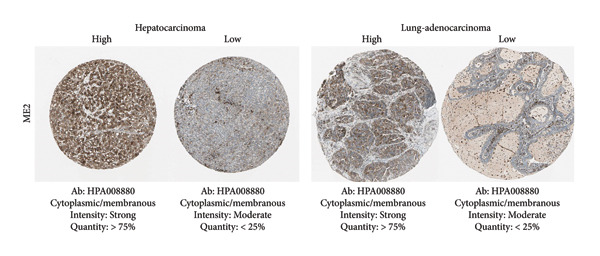
(d)

Figure 2Protein expression patterns of NADPH‐related enzymes that exhibited low‐expression associations with malignancy and cancer prognosis: (a) GDH, (b) ALDH1L1, (c) ALDH1L2), or inconclusive expression pattern (d) DHFR. The immunostaining was predominantly cytoplasmic and membranous. The immunohistochemistry images were obtained from the protein atlas database (https://www.proteinatlas.org) and are shown exclusively for illustrative purposes; clinical annotations and survival data for the corresponding cases are not available. The antibodies (Ab) used were validated and reported by the database, and their reference numbers are indicated below each image. The biomarker is indicated on the left side of the image. The expression was assessed based on the intensity of staining (weak, moderate, and strong) and the number of positive cells (negative, < 25%, 25%–75%, and > 75%), and the expression was classified as high, medium, or low.

**Figure:**
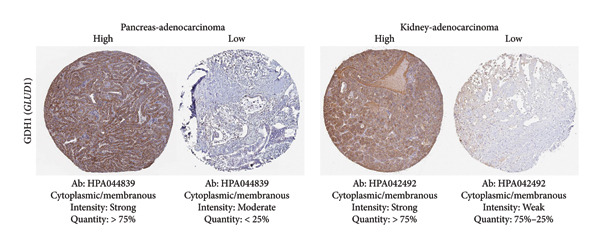
(a)

**Figure:**
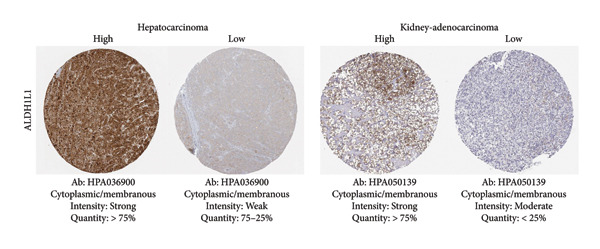
(b)

**Figure:**
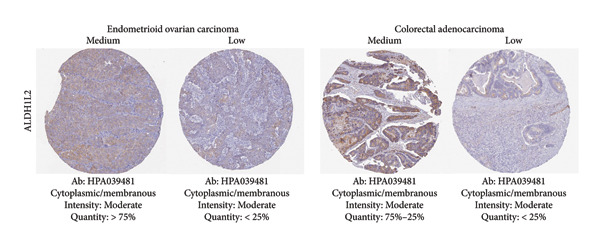
(c)

**Figure:**
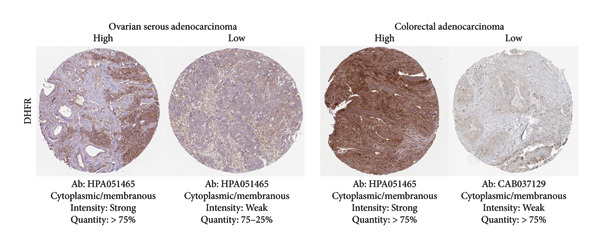
(d)

This review summarizes evidence regarding the protein expression of NADPH‐related enzymes and their associations with prognostic features of cancer. The most relevant enzymes are discussed in the following topics, emphasizing their immunostaining application. Studies exploring NNT and NADK expression in cancer are still scarce, whereas IDH1/2 have been extensively investigated in central nervous system tumors and leukemias [59] with a huge number of publications. These three enzymes require further attention and have not been addressed in this study.

## 2. G6PD and MTHFD Family

**Figure 3:**
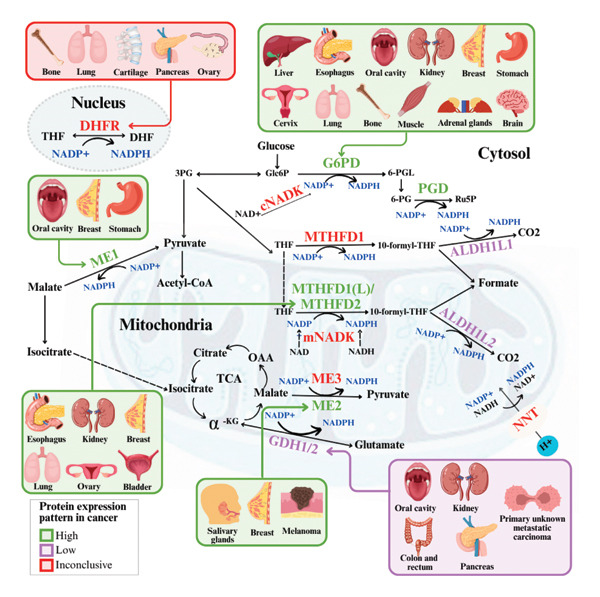
Graphical depiction of the metabolic pathways from the cytosolic (G6PD, PGD, ME1, MTHFD1, and ALDH1L1), mitochondrial (ME2/3, MTHFD1L, MTHFD2, ALDH1L2, GDH1/2, mNADK, and NNT), and nucleic (DHFR) NADPH‐related enzymes. G6PD, MTHFD2, ME1, ME2, and GDH1/2 protein expression exhibited associations with malignancy or prognostic factors in different types of cancer (outlined in green or purple). The DHFR IHC profile was inconclusive regarding malignancy or prognostic factors across distinct primary tumors (outlined in red). The figure was created using the online tool https://www.canva.com/.

NADPH production in cancer cells is facilitated by augmented glucose flux through the pentose phosphate pathway (PPP), essential for nucleotide and lipid biosynthesis and redox balance. Among PPP enzymes, the G6PD functions as a rate‐limiting enzyme by dehydrogenating Dehydrogenating glucose‐6‐phosphate (G6P) to produce 6‐phosphogluconolactone and NADPH, whereas PGD generates ribulose‐5‐phosphate and a second NADPH [7, 20, 21]. G6PD elevated expressions have been reported in different human cancer (Figure 3), including adrenal carcinoma [25], hepatocellular carcinoma [11–13], and cervical cancer [20], where levels were higher than those in non‐neoplastic and benign tissues. G6PD downregulation impairs cell proliferation and promotes apoptosis in HPV18+ HeLa cells [20], whereas reduces invasion, migration, and Hepatitis B virus replication in liver cancer cell lines [11].

In breast cancer, G6PD expression correlates with molecular subtypes, N stage, tumor grade, metastasis, curative status postneoadjuvant chemotherapy, progression‐free, and overall survival. Higher expression was found in Luminal A and B tumors compared to triple‐negative and Human epidermal growth factor Receptor 2 (HER2) positive groups [15], being more pronounced in metastatic lesions [14]. Positive staining in brain metastases correlated with HER2 amplification and Luminal B subtype, albeit without prognostic significance [21]. Increased G6PD was reported in RCC [17], indicating tumor extension, lymph node metastasis, Fuhrman grade, Tumor, node, metastasis classification system (TNM stage), and poor overall survival for clear‐cell RCC [60], whereas the gene expression was associated with advanced Fuhrman grade and tumor aggressiveness [16]. Intriguingly, in vitro studies showed that G6PD knockdown with shRNA reduces NADPH and ROS levels in a NOX4‐dependent manner [61], but inhibition with 6‐Aminonicotinamide (6AN) in primary renal tumor cells decreases NADPH and raises ROS [62]. Thus, depending on the experimental model, the antitumor effects of G6PD suppression may be independent of ROS production [63–66].

The prognostic potential of G6PD immunostaining extends to oral squamous cell carcinoma [23], esophageal [19], gastric [18], and lung cancers [22], where protein and mRNA increased levels were found in advanced TNM stage, lymph node involvement, and distant metastasis, predicting shorter overall survival. G6PD suppression, in animal model of oral carcinoma, impairs lymphatic metastasis via c‐Jun N‐terminal kinase (JNK) pathway, and JNK inactivation reverses the effects of G6PD knockdown on migration, invasion, and epithelial–mesenchymal transition [23]. Conversely, reduced G6PD activity occurs in nasopharyngeal cancer [67] and acute lymphocytic leukemia [68]. In mesenchymal tumors, high G6PD protein was found in 90% of pediatric rhabdomyosarcoma cases, but only in 45% of recurrent tumors [24], suggesting that PPP is mainly involved in primary tumor development than in tumor relapse. G6PD overexpression was also reported in osteosarcoma without clinical or pathological investigations [26]. Experimental findings support the oncogenic potential of G6PD, since its overexpression alters cell morphology, disrupts contact inhibition, and promotes rapid fibrosarcoma growth in mice [69].

In hepatocellular carcinoma, G6PD was integrated into a prognostic model validated by qRT‐PCR and IHC [12]. Intriguingly, inhibition with dehydroepiandrosterone (DHEA) suppresses the proliferation of preneoplastic lesions without affecting NADPH levels, while prolonged treatment induces hepatocarcinogenesis through DNA synthesis and c‐fos/c‐Ha‐ras expression [70, 71].

Mitochondrial one‐carbon metabolism is another major NADPH source, where the MTHFD family (MTHFD1, MTHFD1L, MTHFD2, and MTHFD2L) supports DNA synthesis and redox control. MTHFD1 and MTHFD2 catalyze the conversion of 5,10‐Methylene‐tetrahydrofolate (5,10‐methylene‐THF) to 10‐Formyl‐tetrahydrofolate (10‐formyl‐THF), while MTHFD1L produces formate and generate NADPH [72, 73]. Regarding the protein levels in tumor specimens, MTHFD1 high expression was associated with a poor prognosis and time to recurrence in hepatocellular carcinoma, suggesting a tumor‐promoting role [27]. Conversely, experimental models showed that MTHFD1 deficiency may exacerbate chemical‐mediated tumorigenesis in colon [74]. Additionally, MTHFD1 was detected in both gallbladder carcinoma and non‐neoplastic tissues, with no significant differences between the groups [28]. These observations reflect the context‐dependent effects of MTHFD1 in cancer, with its role varying according to tumor type and metabolic environment. Furthermore, the scarcity of studies still limits its potential as biomarkers.

In contrast, MTHFD2 has emerged as a key oncogenic factor. It is expressed in embryonic tissues and absent in most differentiated adult tissues, presenting robust overexpression in lung [9], breast [33], esophageal [36], ovarian [37], and renal cancers [34, 35], where the strong immunostaining was associated with TNM stage, histological grade, lymph node metastasis, Fuhrman grade, and poor overall survival. MTHFD2 protein expression was also higher in bladder tumors compared to non‐neoplastic tissues but without association with disease progression [38]. In oral cancer, the gene expression of *MTHFD1*, *MTHFD1L*, and *MTHFD2* both distinguished tumor from non‐neoplastic samples, and the overexpression was related to a worse prognosis [75]. In vitro studies revealed that MTHFD2 modulates redox homeostasis and induces cell proliferation independently of metabolic functions [76]. Moreover, MTHFD1/2 knockdown exerts stronger antiproliferative effects in adenocarcinoma than in small‐ and squamous‐cell lung cancer [9].

MTHFD1L supports purine and thymidylate synthesis, acting in cell proliferation, migration, and apoptosis functions of different types of cancer [30, 31, 77]. The protein overexpression was related to malignancy in hepatocellular [32] and esophageal cancer [30], whereas in colorectal tumor [31], it was an adverse prognosis factor associated with tumor grade, TNM stage, invasion, risk of death, and metastasis. The gene expression predicts vital status and/or overall survival for hepatocellular carcinoma [32] and early breast cancer [77]. While high serum MTHFD1L levels were reported in melanoma patients, especially with large tumors (T3/T4) and recurrence, the tissue staining showed a limited prognostic value [29].

Together, G6PD and MTHFD family members exemplify key NADPH‐generating enzymes that integrate metabolic reprogramming with tumor aggressiveness. Their dual roles in anabolic growth and redox maintenance underscore promising opportunities for precision oncology as prognostic biomarkers and therapeutic targets.

## 3. MEs

The MEs (ME1, ME2, and ME3), although structurally similar proteins involved in mammalian metabolism and differentiation, exhibit distinct roles in cancer progression. They catalyze malate oxidative decarboxylation to pyruvate while reducing NAD(P) + to NADPH. ME1 is crucial for NADPH production in the liver and lipid cells during fatty acid biosynthesis and contributes to cancer cell proliferation, mobility, and lactate production [78, 79]. IHC findings indicate that high ME1 levels correlate with a poor prognosis in breast [41], gastric [40], and oral cancers [39]. In breast, it was associated with tumor size, lymph node metastasis [41], and recurrence [80]. In oral cancer, it also predicted size and nodal involvement [39]. *ME1* gene expression was identified as a prognostic factor in breast [41] and laryngeal tumors [81]. Notably, ME1 overexpression can result from p53 mutation, shifting cell metabolism toward aerobic glycolysis [39, 82].

ME2 generates Nicotinamide Adenine Dinucleotide (NADH) and NADPH in mitochondria, sustaining lipid synthesis and glutathione reduction. Since cancer cells use glutamine, glutamate, and pyruvate as substrates, high ME2 levels rewire metabolism to favor growth [83]. *ME2* gene overexpression has been reported in melanoma [42] and erythroleukemia [84], while immunostaining associates it with malignancy in salivary gland tumors [43] and melanoma [42]. In breast cancer, high ME2 protein correlates with nodal metastasis, pathological staging, and vascular embolus [44]. Experimental models of breast cancer [44] and melanoma [42] support ME2’s role in metastasis, anchorage‐independent cell growth, proliferation, and ROS neutralization. Studies in rat epithelial and hepatoma cells showed rapid mitochondrial ME activity during tumor progression [85]. Like ME1, ME2 is negatively regulated by p53 and establishes a feedback loop between p53‐dependent senescence and ME2‐mediated metabolic rewiring [82].

ME3 is expressed in muscles and brain, where it supports mitochondrial fatty acid biosynthesis [79, 86], but its role in cancer is poorly understood. A robust study on gastric cancer found low ME3 expression associated with adverse prognostic factors, including lymph node metastasis, TNM stage, and survival [87]. ME3 knockdown enhanced malignant phenotype by decreasing α‐ketoglutarate (α‐KG) and NADPH/NADP + ratios and increasing malate and Adenosine triphosphate (ATP) under normoxia. Hypoxia further reduced α‐KG and NADPH/NADP + while elevated ROS, suggesting that ME3 downregulation drives gastric cancer progression via redox imbalance and hypoxia‐dependent mechanisms [87].

In summary, MEs, through their isoform‐specific contributions to metabolic rewiring, play multifaceted roles in tumor progression, with ME1 and ME2 emerging as consistent prognostic factors, while ME3 requires further investigation.

## 4. GDHs

The GDH enzyme participates in catabolic and anabolic reactions, catalyzing the reversible conversion of glutamate to α‐KG and ammonia while reducing NAD(P) + to NADPH [88]. In humans, GDH1 and GDH2 are encoded by *GLUD1* and *GLUD2.* Their activities are regulated by inhibitors such as Guanosine triphosphate (GTP) and ATP, or activators such as Guanosine diphosphate (GDP), Adenosine diphosphate (ADP), and leucine [89]. IHC‐positive expression was reported in non‐neoplastic tissues, including liver, pancreas, renal cortex, and testis [90]. Punctate/reticular GDH1 labeling and diffuse GDH2 staining in kidney tubules suggest roles in proton excretion and acid–base balance [91].

Few studies described low GDH protein expression related to cancer aggressiveness, including larger pancreatic tumors [47] and poor survival in metastatic tumor [45]. Reduced protein levels were also observed in a small cohort of RCC compared to non‐neoplastic tissues [49]. By the *in silico* analysis, decreased *GDH* mRNA was correlated with tumor recurrence, advanced stage, Fuhrman grade, and diminished sensitivity to tyrosine kinase inhibitors (TKI). Notably, its overexpression inhibited cell proliferation and migration by suppressing mTOR pathway in renal cancer [49]. The interaction between mTOR and glutamine metabolism regulate cell growth and death, with mTOR indirectly activating GDH [92, 93].

Contrasting, in colorectal cancer [46], high GDH protein was associated with tumor size, advanced stages, and metastasis, suggesting it as an independent prognostic factor. In oral lesions [48], GDH1/2 showed stronger labeling in intraepithelial neoplasia and invasive tumors compared to non‐neoplastic samples. In glioblastoma, the higher immunostaining was associated with a prognosis and, glucose deprivation induced GDH1 phosphorylation (pS384) and Nuclear factor kappa‐light‐chain‐enhancer of activated B‐cells (NF‐κB) activation, enhancing glucose uptake and survival, while GDH1 depletion impaired viability only under low glucose [94]. Under glucose availability, cancer cells downregulate GDH to favor nucleotide and antioxidant synthesis [95], which partially explain the low expression observed in specific tumor types. Briefly, the GDH exhibits context‐dependent roles in cancer, with either reduced or elevated expression influencing prognosis and tumor behavior, highlighting its regulation by metabolic and nutritional status.

## 5. ALDH1Ls

Two members of the ALDH1L family, also known as 10‐formyltetrahydrofolate dehydrogenase, are relevant in cancer studies. ALDH1L1 is a cytosolic folate‐metabolizing enzyme that removes excessive one‐carbon units, limiting their flux toward biosynthetic reactions required for cell proliferation. Acting as a tumor suppressor, ALDH1L1 inhibits proliferation and motility and induces apoptosis [50, 96]. In hepatocellular carcinoma, low ALDH1L1 protein expression was associated with tumor differentiation, HBsAg status, serum AFP, and poor overall survival, with lower mRNA levels in tumors compared to non‐neoplastic tissues [50]. In oral carcinoma, reduced protein levels correlated with recurrence and survival, whereas protein and mRNA expression suggested T3/T4 stages, advanced grades, and high Ki‐67. Although ALDH1L1 catalyzes NADPH‐generating reactions, its downregulation does not initiate carcinogenesis but confers metabolic advantage for tumor growth through PI3K–Akt–Rb signaling rather than redox regulation. Its contribution to antioxidant defense appears limited compared with the mitochondrial homolog ALDH1L2 [51, 97].

ALDH1L2 also catalyzes 10‐formyl‐THF hydrolase reactions and contributes to NADPH generation, mitochondrial protein biosynthesis, and CoA‐dependent pathways. In colorectal cancer [52], low ALDH1L2 protein expression was associated with poor disease‐free survival and inversely correlated with tumor regression score. Reduced mRNA levels were found in patients unresponsive to neoadjuvant chemoradiotherapy, whereas protein expression indicated radioresistance. Since ALDH1L2 regulates NF‐κB signaling via thioredoxin (TXN), a redox protein that protects cells from oxidative stress, TXN inhibition with PX‐12 may overcome ALDH1L2‐driven radioresistance [52].

Altogether ALDH1L1 and ALDH1L2 downregulation consistently suggests tumor aggressiveness and therapy resistance, underscoring their relevance as prognostic biomarkers and potential therapeutic targets. However, their effects appear tumor specific, and the precise mechanisms underlying their role in tumor progression remain to be elucidated.

## 6. DHFR

The DHFR plays a critical role in DNA synthesis in eukaryotic and prokaryotic cells. It reduces folic acid to dihydrofolic acid and converts dihydrofolate to tetrahydrofolate (THF), which participates in purine, methionine, thymidylate, and amino acid production. Within this context, NADPH serves as an electron donor to catalyze DHFR reduction reactions [98, 99]. Given its role in cellular proliferation, DHFR has been considered a therapeutic target for cancer, bacterial infections, and parasitosis [100].

Although the DHFR expression varies across cancer types, its gene overexpression was implicated in an unfavorable prognosis for brain tumors and leukemia [101, 102]. In osteosarcoma, the positive immunostaining has been correlated with relapses and poor outcomes in patients receiving methotrexate (MTX) chemotherapy [54], a known DHFR inhibitor [98]. Increased protein staining in chondrosarcomas was associated with tumor severity, progression, and poor prognosis, suggesting DHFR as a malignancy biomarker [56]. In ovarian cancer, western blot analysis of 160 samples [53] revealed reduced DHFR in malignant tumors, supported by immunoreactivity associations with metastasis, therapy resistance, and prognostic implications. Conversely, DHFR protein profile showed a limited prognostic value in pancreatic [55] and lung cancers, despite its upregulation in tumor recurrence [58]. Another report on lung cancer [57] described minimal DHFR expression in squamous cell carcinoma but strong expression in adenocarcinoma. Moreover, the study suggested a potential association between high expression of thymidylate synthase (TS) and DHFR with reduced disease‐free survival, but the significance remains debatable.

Despite insufficient evidence to advocate the use of DHFR as a prognostic biomarker, it seems promising for evaluating therapeutic response, particularly to MTX and its derivatives, which have been incorporated into anticancer therapy since 1940s [99, 100]. Although reduced DHFR expression may suggest favorable response to pemetrexed‐based chemotherapy, specifically for lung cancer, this association was not consistent through protein and mRNA tissue investigations [103]. Interestingly, low plasma DHFR mRNA expression emerged as a potential strategy to select responsive patients for pemetrexed‐based therapy [104]. In vitro DHFR high levels attenuate tumor cell death and mitigate T‐cell‐mediated antitumoral activity following MTX treatment, while its inhibition reduces cell viability under T‐cell coculture [105]. Overall, DHFR expression exhibits tumor‐type variability, with stronger clinical significance in therapy response than in the prognosis, reinforcing its potential as a target for antifolate‐based strategies.

## 7. Conclusions, Limitations, and Prospects

IHC is a widely accessible method for evaluating cancer biomarkers and can provide relevant information regarding tumor behavior. Despite advances, immunohistochemical studies on NADPH‐related enzymes remain largely unexplored. Among NADPH‐related enzymes, G6PD is notable regarding cancer aggressiveness, especially in hepatocellular, breast, and renal carcinomas. G6PD protein overexpression, as well as MEs and MTHFD2, are associated with tumor progression and represent potential therapeutic targets across various tumor types. This review did not address NNT and IDH1/2 in detail due to a lack of data on NNT protein expression in cancer and, in the case of IDH1/2, due to the huge number of published studies that warrant a specific and more comprehensive review of these biomarkers.

Associations between NADPH‐related enzymes and tumor progression vary according to cancer type, reflecting differences in metabolic reprogramming and the tumor microenvironment. This variability represents both a challenge and an opportunity for their clinical use as biomarkers, since their prognostic or predictive value depends on tumor type or subtype. We emphasize the need for studies focusing on standardizing detection methods for specific tumor entities and that integrate validation cohorts from different locations. Further inquiries, particularly into G6PD and MTHFD2, are essential to confirm potential applications in cancer prognosis and therapy. Moreover, it would be valuable to investigate some of the NADPH‐related enzymes as potential immunohistochemical biomarkers for therapy responses.

**Nomenclature**
10‐formyl‐THF: 10‐Formyl‐tetrahydrofolate
5,10‐methylene‐THF: 5,10‐Methylene‐tetrahydrofolate
NADPH: Nicotinamide adenine dinucleotide phosphate
ADP: Adenosine diphosphate
ATP: Adenosine triphosphate
ALDH1L1 and ALDH1L2: Aldehyde dehydrogenases
c‐Ha‐ras: Cellular Harvey rat sarcoma viral oncogene homolog
c‐fos: Cellular proto‐oncogene Fos
JNK: c‐Jun N‐terminal kinase
DHEA: Dehydroepiandrosterone
G6P: Dehydrogenating glucose‐6‐phosphate
DHFR: Dihydrofolate reductase
G6PD: Glucose‐6‐phosphate dehydrogenase
GDH1 and GDH2: Glutamate dehydrogenases
GDP: Guanosine diphosphate
GTP: Guanosine triphosphate
HBsAg: Hepatitis B surface antigen
HER2: Human epidermal growth factor Receptor 2
IHC: Immunohistochemistry
ME1, ME2, and ME3: Malic enzymes
MMP2: Matrix Metalloproteinase 2
MTX: Methotrexate
MTHFD1L: Methylene tetrahydrofolate dehydrogenase 1‐like
MTHFD1, MTHFD1L, MTHFD2, and MTHFD2L: Methylenetetrahydrofolate dehydrogenases
MAPK: Mitogen‐activated protein kinase
NADK: Nicotinamide adenine dinucleotide kinase
NADP+: Nicotinamide adenine dinucleotide phosphate (oxidized)
NADPH: Nicotinamide adenine dinucleotide phosphate (reduced)
NNT: Nicotinamide nucleotide transhydrogenase
NMDMC: NAD‐dependent mitochondrial methylenetetrahydrofolate dehydrogenase‐cyclohydrolase
NF‐κb: Nuclear factor kappa‐light‐chain‐enhancer of activated B‐cells
PPP: Pentose phosphate pathway
PGD: Phosphogluconate dehydrogenase
qRT–PCR: Quantitative reverse transcription polymerase chain reaction
ROS: Reactive oxygen species
RCC: Renal cell carcinoma
serum AFP: Serum alpha‐fetoprotein
THF: Tetrahydrofolate
TXN: Thioredoxin
TS: Thymidylate synthase
TCA cycle: Tricarboxylic acid cycle
p53: Tumor Protein p53
TNM stage: Tumor, node, metastasis classification system
TKI: Tyrosine kinase inhibitor
WHO: World Health Organization
α‐KG: α‐Ketoglutarate

## Ethics Statement

The authors have nothing to report.

## Conflicts of Interest

The authors declare no conflicts of interest.

## Author Contributions

Camila M. Scudeler: methodology, literature search and curation, writing–original draft preparation, and critical review and editing. Keila Samantha da Silva: methodology, literature search and curation, and writing–original draft preparation and editing. Rafaela V. N. Silva: writing–review and editing. Juliana Reis Machado: writing–original draft critical review. Adilha M. R. Micheletti: writing–original draft critical review. Karen B. Ribeiro: writing–original draft preparation and critical review and editing. Régia C. P. Lira: conceptualization and idea for the article, supervision, literature curation, writing–original draft preparation, and critical review and editing.

## Funding

This study is part of a research funded by the *Fundação de Amparo à Pesquisa do Estado de Minas Gerais*—FAPEMIG (Protocol number: APQ‐00258‐21).

## Data Availability

Data sharing is not applicable to this article as no new data were created or analyzed in this study.
